# Manipulation of metabolic pathways to promote stem-like and memory T cell phenotypes for immunotherapy

**DOI:** 10.3389/fimmu.2022.1061411

**Published:** 2023-01-18

**Authors:** Michael D. Claiborne

**Affiliations:** Department of Medicine, Scripps Green Hospital and Scripps Clinic, La Jolla, CA, United States

**Keywords:** immunology, T cell memory, metabolism, tumor immunology, CAR T cancer therapy, adoptive cell immunotherapy

## Abstract

Utilizing the immune system’s capacity to recognize and kill tumor cells has revolutionized cancer therapy in recent decades. Phenotypic study of antitumor T cells supports the principle that superior tumor control is achieved by cells with more long-lived memory or stem-like properties as compared to terminally differentiated effector cells. In this Mini-Review, we explore recent advances in profiling the different metabolic programs that both generate and define subsets of memory T cells. We additionally discuss new experimental approaches that aim to maximize the durability and sustained antitumor response associated with memory T cells within the unique immunosuppressive conditions of the tumor microenvironment, such as engineered attempts to overcome hypoxia-induced changes in mitochondrial function, the inhibitory effects of tumor metabolites, and exploitation of more recently-defined metabolic pathways controlling T cell memory fate such as glycogen metabolism.

## Introduction

Exploitation of the immune system’s ability to target and kill tumor cells has demonstrated profound clinical benefit for a multitude of cancer types in the form of checkpoint blockade, whereby blocking inhibitory signaling molecules on T cells allows for the host or patient’s own immune system to mount an effective antitumor T cell response (1). A related avenue of investigation involves adoptive cellular therapies (ACT) for cancer, involving the transfer of tumoricidal immune cells that can be modified *ex vivo*, such as chimeric antigen receptor T cell (CAR T cell) or T cell receptor therapy (TCR-T) (2). Analysis of outcomes in checkpoint blockade therapy and ACT have demonstrated superior responses in patients with more detectable stem-like or memory T cell populations (3–5). Phenotypes of responding immune cells in these settings are detailed in additional reviews (6, 7). Immune cell metabolism is increasingly recognized as more than a simple consequence of cellular activity, but rather it defines cellular activity, including memory T cell function (8). However, the development and maintenance of this metabolic state faces unique challenges within the tumor microenvironment (TME), including nutrient deprivation, inhibitory signaling, and the presence of immunosuppressive metabolites (9, 10). In this Mini-Review, we focus on hallmarks of memory T cells that facilitate the metabolism necessary for their identity and function as long-lived cells capable of self-renewal and rapid reactivation upon rechallenge. We review recent advances in defining the many forms of metabolic inhibition imposed upon T cells within the TME. We additionally profile new experimental approaches that aim to manipulate the metabolic properties of T cells to support stem-like or memory phenotypes, with the goal of maintaining these phenotypes within the TME to achieve tumor control.

## Metabolic shifts in T cell activation and differentiation

Naïve T cells primarily rely on oxidative phosphorylation to maintain energy homeostasis prior to antigen encounter, but undergo radical metabolic changes upon TCR activation, with glycolysis and glutaminolysis fueling effector responses characterized by cytokine production and clonal expansion (11). Pro-growth signaling pathways downstream of TCR signaling such as the PI3K/Akt/mTOR pathway further support these metabolic changes by promoting expression of transcription factors such as c-Myc, which controls cell cycle entry in addition to amino acid and glucose uptake (12, 13). Glycolysis is additionally sustained by the downstream effects of CD28 costimulation, which further maintains PI3K/Akt/mTOR-mediated GLUT1 mobilization for continued glucose uptake (14). Although most clonally-expanded effector T cells die off over the course of antigenic response, a small percentage persist and become long-lived memory T cells through mechanisms still under investigation (15). Effector memory (Tem) cells are distinguished from central memory (Tcm) cells by greater cytokine production but less long-term persistence. Memory T cells display lower baseline activity of anabolic signaling pathways such as PI3K-Akt-mTOR (16). Consistent with the lack of signaling and machinery supporting constitutive glycolysis, they instead upregulate surface transporters for fatty acid uptake in an IL7R- (CD127)-dependent manner as well as mitochondrial enzymes responsible for fatty acid oxidation (17). More recently, a subset of T cells termed stem cell memory (Tscm) have been defined by expression of markers such as CD95 and CXCR3 in addition to CD127 that maintain the greatest proliferative potential among memory T cells and can in fact reconstitute an entire host’s T cell compartment, including Tcm and Tem cells (18, 19). Components of memory T cell metabolism that permit for rapid reacquisition of effector function upon reactivation are detailed in the next section.

The majority of research in T cell metabolism has been conducted using T cells activated and cultured *in vitro*. As metabolism across cell types can vary widely by tissue microenvironment, this raises the question of the generalizability of these studies to T cell metabolism *in vivo*. Indeed, recent studies comparing the metabolism of T cells activated *in vivo* or *in vitro* in viral infectious models have called into question the extreme shift towards glycolysis in effector cells, as Ma et al. demonstrated that *in vivo-*activated CD8+ T cells maintain high rates of mitochondrial respiration with increased spare respiratory capacity (SRC) when compared to *in vitro*-activated cells. In fact, in these studies, T cells at the peak of effector responses exhibited a metabolic profile much more similar to naïve cells in terms of energy metabolism (20). However, the contribution of glycolysis to proliferative capacity in these cells was critical not as it related to ATP generation, but rather through glucose-dependent serine biosynthesis. Additional studies using media with physiologic carbon sources (PCS) found in animal serum but less abundant in traditional cell culture media such as acetate and β-hydroxybutyrate have demonstrated that metabolites such as lactate, once thought to be largely immunosuppressive, function as critical TCA cycle fuel for T cells activated under more physiological metabolic conditions in infectious models (21). These studies highlight the importance of methodology when studying metabolic processes and the need for future studies to further characterize the activation conditions of experimental T cells, as this has profound implications for future cellular metabolic function.

Once a population of memory T cells is established, multiple factors maintain their identity and function. We next briefly review the factors that maintain the metabolic state of memory T cells. This does not serve as an exhaustive list, and the many aspects of metabolism which control the identity of memory T cells are further explored in other reviews (22). Rather, this list serves to outline specific factors discussed in later sections with therapeutic implications in the tumor microenvironment.

## Regulation of memory T cell metabolic state

Epigenetic control of metabolism as it pertains to memory cells is a consequence of the integration of environmental signals from the time of T cell activation onward, as metabolites themselves can directly or indirectly function in both DNA and histone modification programs that support effector or memory fates. For example, elevated acetate levels in T cells at the peak of effector responses facilitate GAPDH acetylation (leading to enzymatically increased glycolytic activity) while simultaneously increasing H3K9 acetylation at loci critical for future effector function (23, 24). Subsets of dividing cells experience differential histone modification depending on cellular fate, as demonstrated when comparing populations of cells with differential IL7R expression, a classic marker of long-lived T cells. IL7R^low^ terminally differentiated effector T cells begin to demonstrate increased transcriptional silencing at loci critical to memory function in the form of H3K27 trimethylation compared to IL7R^hi^ effector memory cells (25). As it pertains to DNA modification, distinct patterns of demethylation of effector loci have been observed in T cells as they progress to more central memory or stem-like memory fates that facilitate rapid reacquisition of effector function (26). The multitude of factors that control epigenetic fate in developing T cells are explored in-depth in other reviews (27, 28). As it pertains to this review, modifications to DNA and histones that facilitate the metabolism necessary to support both a rapid recall response and enhanced survival within the tumor microenvironment are of interest, as interventions there have direct implications in cellular therapies for cancer.

Mitochondrial parameters such as overall mass, SRC, and transmembrane potential serve in shaping T cell fate through the mitochondria’s functional connection to energy metabolism. Memory T cells demonstrate significantly increased mitochondrial mass compared to effector cells, concomitant with increased reliance on oxidative phosphorylation for energy production (16, 29). Excessive reactive oxygen species (ROS) production as a result of the biochemistry of oxidative phosphorylation can become detrimental to T cells, demonstrated by the predictive ability of mitochondrial transmembrane potential (ΔΨm) in determining future *in vivo* antitumor activity. T cells separated by higher baseline transmembrane potential demonstrated lower SRC, greater intracellular ROS production, and an increase in expression of effector-related genes with decreased expression of memory-related genes compared to cells with lower transmembrane potential (30). Ongoing research continues to clarify these and other properties, such as mitochondrial fusion, in T cell memory function (31). For the purpose of this review, we will focus on dysregulation of oxidative phosphorylation and inability to moderate ROS production in the TME as parameters of mitochondrial metabolism amenable to therapeutic intervention.

The role of various cytokines both in T cell priming and in the TME can have profound implications in memory function. IL-2, IL-7, and IL-15 are members of the common gamma chain family of cytokines with differential effects on T cell function. IL-2 functions to stimulate the PI3K-Akt-mTOR pathway, stabilize HIF1α, and facilitate glucose uptake, while IL-7 is critical for memory T cell survival *via* regulation of glycerol uptake and movement of fatty acids into the mitochondria *via* CPT1α (17, 32, 33). IL-15 signaling plays roles in homeostatic proliferation of memory T cells and primes them for cytokine re-expression upon rechallenge (34, 35). As will be discussed later, treatment of antitumor T cells with IL-7 and IL-15 have shown promise in increasing parameters associated with memory or stem-like phenotypes. Conversely, cytokines such as TGF-β encountered in the TME have broad immunosuppressive or immune-exclusionary functions and can directly impair T cell responses through Smad-mediated downregulation of critical genes such as granzyme and perforin (36, 37). We next review additional metabolic contributors within the TME, as their study is essential to understanding and improving antitumor immune responses.

## T cell metabolism in the TME

The nutrient-deprived, acidic, hypoxic conditions encountered in solid tumors pose unique challenges to tumor infiltrating lymphocytes (TILs). Nutrient competition, whereby tumor cells ultimately prevail over immune cells for critical molecules such as glucose and methionine, represents a major mechanism of immunosuppression (38, 39). Expanding this concept of competition in the TME to entire organelles, Saha et al. recently described a nanotubule-mediated process by which tumor cells steal mitochondria from infiltrating immune cells, rendering them less energetically fit (40). Tumor-derived lactate not only suppresses cytotoxic activity *via* mechanisms including inhibition of glycolysis and concomitant serine biosynthesis *via* disruption of NAD+/NADH redox balance, but has been recently demonstrated to serve as fuel for metabolically-flexible regulatory T cells (Tregs) within the tumor microenvironment, further suppressing antitumor immune responses (41–44). Additional immunosuppressive metabolites such as adenosine and kynurenine further decrease T cell responsiveness by suppressing TCR signaling, inhibiting cytokine production and stabilizing the defining Treg transcription factor Foxp3 in the case of adenosine and by stimulating Treg differentiation and increasing T cell expression of inhibitory checkpoint molecules such as PD-1 in the case of kynurenine (45–47). Tumor-derived retinoic acid stimulates differentiation of monocytes into a more immunosuppressive and less pro-inflammatory phenotype, suppressing T cell responses (48). Extracellular potassium, increased in the TME as a consequence of cellular necrosis, serves to induce T cell metabolic programs that restrain acquisition of effector function *via* decreases in acetyl-CoA-mediated epigenetic remodeling, yet also sustains expression of stemness markers such as *Tcf7* (49, 50).

T cell exhaustion is broadly defined as a state of hyporesponsiveness seen in the context of persistent stimulation such as chronic viral infection or cancer and is the subject of existing reviews (51). As it pertains to metabolism, impairment in mitochondrial oxidative phosphorylation with excessive mitochondrial ROS production observed in exhausted T cells appears to be a major driver of T cell dysfunction (52). This impairment appears most pronounced in the setting of hypoxia, as Scharping et al. demonstrated that while chronic stimulation or hypoxia alone generated functional CD8+ effector cells during differentiation, the combination of both factors readily generated extensive mitochondrial ROS and produced exhausted cells (53). Hypoxia, as is often encountered in the TME, has profound effects on previously mentioned suppressive pathways as well, ranging from the stabilization of factors involved in generating extracellular adenosine to downregulation of nutrient uptake transporters critical to metabolic pathways downstream of CD8+ activation (54, 55). Combating T cell exhaustion is central to metabolic interventions in cancer immunotherapy; as discussed in the next section, many innovative approaches to prime or enhance CD8+ T cell memory directly aim to counteract the changes seen in the TME that have been previously summarized.

## Enhancing therapy through metabolic manipulation

Culturing antitumor T cells or CAR-T cells in IL-7 or IL-15 rather than IL-2 alone has been demonstrated to produce cells with greater *in vivo* tumor control and greater preservation of Tscm phenotype (56–58). Benefits of IL-7 or IL-15 exposure extend beyond the T cell priming phase, as engineered modifications to these cytokines improving their *in vivo* persistence have recently produced viable adjuvants that improve antitumor responses when administered to animals in the case of IL-7 and in clinical trials for IL-15 (59, 60). Li et al. recently demonstrated an enhancement of metabolic flexibility with transgenic IL-7 production in CAR T cells, with CD4+ IL-7 CAR T cells demonstrating less overall metabolic activity at rest compared to standard CARs but were capable of rapidly downregulating CPT1α upon re-engaging tumor cells, facilitating shifts away from mitochondrial metabolism towards glycolysis and rapid effector reacquisition, leading to superior tumor control (61). Both exposure to and cell-autonomous production of these cytokines are active avenues of investigation in improving antitumor responses by facilitating pro-memory signals.

**Figure 1:**
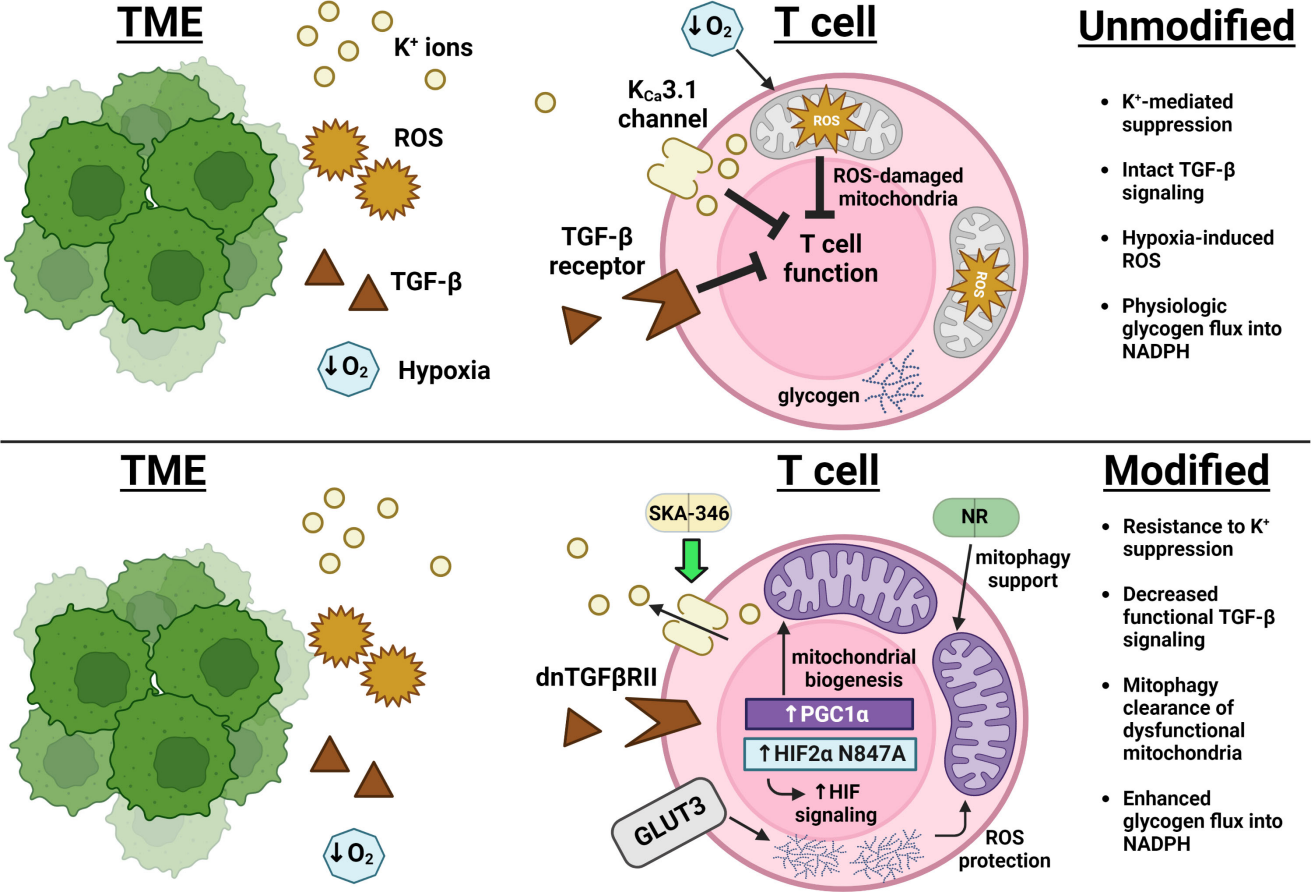
Advances in overcoming immunosuppression and impingement on metabolism in the TME. Excess extracellular potassium (yellow) limits nutrient uptake concomitant with and critical for T cell effector function (49). The K_Ca_3.1 channel activator SKA-346 facilitates extrusion of this potassium to restore production of cytotoxic molecules (50). Expression of a TGF-β receptor mutant that can bind TGF-β but cannot activate SMAD decreases transduction of the broad immunosuppressive signals (brown) downstream of this pathway (36, 37). Overexpression of PGC1α leads to an increase in mitochondrial mass (purple), while overexpression of GLUT3 (gray) supports glucose uptake that fuels glycogen storage, protecting T cells from ROS (orange) encountered both as part of the TME and as a consequence of mitochondrial metabolism (63, 64). Supplementation with nicotinamide ribose (NR) enforces mitophagy of ROS-damaged mitochondria and improves overall mitochondrial fitness (65). Expression of a HIF2α mutant resistant to Factor Inhibiting HIF (FIH)-mediated suppression enhanced T cell responsiveness in the hypoxic TME (blue) (66).

Regarding suppressive signals, Silk et al. have demonstrated a resistance to TGF-β-mediated inhibition of effector function when CAR T cell constructs are expressed alongside a truncated dominant-negative TGF-β receptor (dnTGFβRII), which can bind TGF-β but does not signal through Smad (62; **Figure 1**). Oxygenation supplementation to living organisms through means such as hyperbaric oxygen therapy (HBOT) attempting to decrease TME hypoxia would seem at first glance to comprise a reasonable anticancer therapy. However, most large studies in humans have been inconclusive and both preclinical and clinical studies suggest lack of benefit in some settings but benefit in others (67). For example, Liu et al. recently demonstrated extracellular matrix remodeling of stroma-rich tumors in mice exposed to HBOT, which permitted for greater accessibility of both T cells and immune checkpoint antibodies to the intratumoral space which thereby improved tumor control (68). Restriction of efficacy of this therapeutic modality to only a subset of tumors, such as those with extensive extracellular matrix formation, could explain the lack of consistent human data demonstrating benefit. However, efforts to overcome hypoxia-induced inhibition in a T cell-intrinsic manner have met success with overexpression of a HIF2α mutant resistant to the oxygen-dependent negative HIF regulator Factor Inhibiting HIF (FIH), increasing cytolytic function in CD8+ T cells and improving tumor control in CAR T cell animal models (66). The inhibitory effect of extracellular potassium can pharmacologically abrogated, as treatment of T cells with the K_Ca_3.1potassium channel activator SKA-346 prevents potassium-mediated suppression of cytotoxic function *in vitro* (50, **Figure 1**). In targeting pH balance, genetic deletion of the chloride/bicarbonate anion transporter Ae2 (also known as SLC4A2) which extrudes bicarbonate down physiologic gradients in CD8+ T cells improves T cell cytokine production and increase proportion of memory phenotype cells after activation *in vitro* and improves tumor control in ACT experiments *in vivo* by preventing bicarbonate loss to acidic environments (69, 70). Further characterization of inhibitory signals in the TME will lead to advances in engineering T cell resistance to these forms of suppression.

Manipulation of mitochondrial biogenesis or function has shown promise in improving antitumor responses. Overexpression of PGC1α, the master regulator of mitochondrial biogenesis, increased mitochondrial content, skewed T cells towards a KLRG1^lo^CD127^hi^ Tcm phenotype, and decreased tumor burden in mouse melanoma models (63; **Figure 1**). Addressing CD8+ T cell mitochondrial function, Yu et al. recently described a mitophagy-dependent nicotinamide ribose (NR) supplementation strategy that improves antitumor immunity by preventing accumulation of dysfunctional mitochondria (65). Temporal alterations in mitochondrial metabolism have attracted recent attention, as short-term blockade of mitochondrial pyruvate ingress *via* inhibiting the mitochondrial pyruvate carrier MPC1 in nutrient-rich culture prior to adoptive T cell transfer has been shown to increase uptake of glutamine and fatty acids with subsequent acetyl-CoA generation, permitting for increased H3K27Ac-mediated accessibility of critical memory loci such as *Sell, Ccr7*, and *Tcf7* (71). Wholesale blockade of MPC1 within the nutrient-poor TME, however, led to decreased antitumor T cell responses by inhibiting the ability of T cells to utilize lactate to fuel the TCA cycle, implicating epigenetic modification as a consequence of alterations in mitochondrial metabolism as the major driver of this improved antitumor phenotype.

Upregulated glycogenolysis leading to glucose-6-phosphate-derived antioxidant NADPH generation *via* phosphoenolpyruvate carboxykinase (Pck1) is a recently defined characteristic of memory T cells that protects them from ROS generated during fatty acid oxidation (72). Recently, an epigenetic regulatory mechanism linking Pck1 activity to other aspects of memory T cell metabolism has been proposed by Ma et al., whereby mitochondrial acetyl-CoA in memory cells is diverted to ketogenesis, with subsequent β-hydroxybutyrate production epigenetically modifying H3K9 at loci encoding Foxo1 and the master mitochondrial biogenesis co-activator PGC1α *via* β-hydroxybutyrylation (73). This epigenetic activation not only promotes Pck1 expression, but supports mitochondrial biogenesis as a whole. The contribution of glycogen to memory metabolism is further supported in a study by Criboli et al., where overexpression of the glucose transporter GLUT3 created T cells with increased glucose uptake with subsequent glycogen storage, leading to increased mitochondrial fitness, reduced ROS, and greater tumor control (64; **Figure 1**). Mice in these experiments were protected from tumor rechallenge, suggesting the generation of a potent antitumor immune response. Further modulation of genes in the ketogenesis pathway could thereby potentially alter flux, metabolite availability, and epigenetic control of this key determinant of memory cell metabolism.

Recent work has additionally implicated metabolism of vitamin A metabolites in the epigenetic control of T cell differentiation. Fujiki et al. described T cell-intrinsic metabolism of vitamin A to retinal by the enzyme RDH10, which subsequently downregulated CD62L expression *via* retinoic acid receptor (RAR) signaling leading to H3K27 trimethylation-mediated epigenetic repression of memory-associated loci such as *Tcf7* and *Bcl2*, supporting terminal effector differentiation of activated T cells (74). RDH10-deficient T cells were protected from this forced differentiation in the TME and were more memory-like, controlling tumors to a greater degree than wild-type counterparts. As noted previously, the TME is frequently enriched in tumor-derived retinoic acid (48). While RAR signaling has been demonstrated to play a role in effector T cell responses (75), the optimal duration and strength of this signal to permit antigen responses with subsequent memory generation while preventing terminal differentiation programs remains to be determined. As it pertains to other vitamin families, while less literature exists on their role in preserving a memory phenotype, vitamin E has recently been demonstrated to improve anticancer immune responses by enhancing dendritic cell cross-presentation of tumor antigens in animal models, while analysis of patients undergoing checkpoint blockade therapy revealed increased survival among those taking vitamin E (76). As vitamins are critical cofactors in all cells, additional investigation of their differential effects on tumor and immune cells could uncover pathways that could be manipulated to improve immunotherapeutic responses.

## Conclusion

Parameters associated with memory T cells, such as master regulators of energy metabolism and epigenetic regulation of loci critical to longevity or recall response, have been manipulated in pre-clinical settings with improvements in antitumor response. Future advances in the field will not only include the definition of novel T cell metabolic pathways that define memory responses but also determining outcomes of manipulating multiple nodes of these pathways in therapeutic settings. For instance, how would the balance of mitochondrial ROS production be affected by overexpression of regulators of mitochondrial biogenesis alongside glycogen synthesis upregulation in the context of the epigenetic reprogramming seen under MPC1 inhibition at T cell priming? As an excess of mitochondrial ROS has demonstrated deleterious effects on T cell function (53), balancing these interventions while maximizing the longevity and magnitude of T cell memory responses represents an exciting new avenue in cancer immunotherapy research. Moreover, would multiple altered pathways, such as enhanced glycogen metabolism in the context of resistance to TGF-β signaling, act in an additive or in a synergistic manner as it pertains to enhancement of T cell function with resultant tumor control? Answers to such questions will help to guide the next stages in research and development of cellular therapeutics for cancer. To that end, the activation context of T cells, whether *in vitro* or *in vivo*, serves to dictate their future metabolic function *in vivo* in intriguing and sometimes unexpected ways (20, 21). Incorporating these principles into future metabolic research will be crucial for translating pre-clinical findings into manipulations of T cell function that ultimately would benefit patients.

## Author contributions

MC wrote the manuscript and illustrated the figure. The author confirms being the sole contributor of this work and has approved it for publication.
